# Positive outcomes of oil palm phenolics on degenerative diseases in animal models

**DOI:** 10.1017/S0007114511002133

**Published:** 2011-06-07

**Authors:** Ravigadevi Sambanthamurthi, YewAi Tan, Kalyana Sundram, Kenneth C. Hayes, Mahinda Abeywardena, Soon-Sen Leow, Shamala Devi Sekaran, T. G. Sambandan, ChoKyun Rha, Anthony J. Sinskey, Krishnan Subramaniam, Syed Fairus, Mohd Basri Wahid

**Affiliations:** 1 Malaysian Palm Oil Board, 6, Persiaran Institusi, Bandar Baru Bangi, 43000 Kajang, Selangor, Malaysia; 2 Malaysian Palm Oil Council, 2nd Floor, Wisma Sawit, Lot 6, SS6, Jalan Perbandaran, 47301 Kelana Jaya, Selangor, Malaysia; 3 Brandeis University, 415 South Street, Waltham, MA 02454, USA; 4 Commonwealth Scientific and Industrial Research Organisation, Gate 13, Kintore Avenue, Adelaide, SA 5000, Australia; 5 University of Malaya, 50603Kuala Lumpur, Malaysia; 6 Massachusetts Institute of Technology, 77 Massachusetts Avenue, Cambridge, MA 02139, USA; 7 MAHSA University College, Jalan University Campus, 50603 KualaLumpur, Malaysia

**Keywords:** Oil palm phenolics, CVD, Metabolic syndrome, Anti-tumour mechanisms

## Abstract

It is well established that plant phenolics elicit various biological activities, with positive effects on health. Palm oil production results in large volumes of aqueous by-products containing phenolics. In the present study, we describe the effects of oil palm phenolics (OPP) on several degenerative conditions using various animal models. OPP reduced blood pressure in a NO-deficient rat model, protected against ischaemia-induced cardiac arrhythmia in rats and reduced plaque formation in rabbits fed an atherogenic diet. In Nile rats, a spontaneous model of the metabolic syndrome and type 2 diabetes, OPP protected against multiple aspects of the syndrome and diabetes progression. In tumour-inoculated mice, OPP protected against cancer progression. Microarray studies on the tumours showed differential transcriptome profiles that suggest anti-tumour molecular mechanisms involved in OPP action. Thus, initial studies suggest that OPP may have potential against several chronic disease outcomes in mammals.

Phenolic phytochemicals are secondary metabolites of plant origin. These metabolites protect the plants against biological and environmental stresses. Because of their important protective biological functions, they are ubiquitous in plants and therefore are found in almost all food groups. Despite their wide distribution, the health effects of dietary phenolics have only recently attracted the interest of nutritionists, more attention having been paid before to antioxidant vitamins such as ascorbic acid, tocopherols and carotenoids. The diversity and structural complexity of phenolics is one of the main factors that have delayed their research.

Emerging epidemiological evidence is increasingly attesting to the positive effects of fruits and vegetables in managing chronic and infectious diseases^(^ ^1^ ^,^ ^2^ ^)^. These beneficial effects are attributed to the antioxidant activity of the constituent phenolic metabolites. The phenolic ring and hydroxyl substituents of these compounds can function as effective antioxidants due to their ability to capture free radicals by donating hydrogen atoms or electrons. Their bioactivity may also be related to the ability to chelate metals and inhibit lipo-oxygenases^(^ ^3^ ^)^. However, the biological effects of phenolics may extend well beyond the modulation of oxidative stress. For example, soya isoflavones modulate endocrine function by interacting with oestrogen receptors^(^ ^4^ ^)^. NO bioavailability influences insulin-stimulated glucose uptake and vascular tone^(^ ^5^ ^)^. There is scientific evidence that phenolics exert significant vascular protection because of their antioxidant properties and increased NO bioavailability. A detailed understanding of the molecular events underlying these various biological effects is essential for evaluation of the overall impact on disease risk and progression.

The importance and demand for natural antioxidants have grown in recent years. Fruit processing residues are attractive and inexpensive sources of phenolic antioxidants. For example, apple pomace has been reported to be a source of polyphenolic compounds^(^ ^6^ ^,^ ^7^ ^)^. In the previous study, we showed that the vegetation liquor from the palm oil milling process is a rich source of phenolics and presented evidence based on *in vitro* and *ex vivo* studies that oil palm phenolics (OPP) have free radical-scavenging activity and protective bioactivities against CVD^(^ ^8^ ^)^. In the present study, we describe *in vivo* whole animal studies designed to identify specific bioactivities in relation to chronic diseases. The studies provide an indication that OPP may provide protection against major degenerative disorders including CVD, diabetes and cancer.

## Materials and methods

### Oil palm phenolic samples

For all the animal studies carried out in the present study, OPP was prepared according to the methods described by Sambanthamurthi *et al.* ^(^ ^9^ ^)^. OPP contains numerous phenolic acids. There are three isomers of caffeoylshikimic acid that are found to be major components of the extract^(^ ^10^ ^)^. Other phenolic acids include caffeic acid, protocatechuic acid and *p*-hydroxybenzoic acid. The detailed composition of OPP is as described earlier^(^ ^8^ ^)^.

### Blood pressure studies

The use of animals in the cardiovascular studies (whole animal blood pressure (BP) and cardiac arrhythmia experiments) was approved by the Commonwealth Scientific and Industrial Research Organisation-Health Sciences and Nutrition Animal Experimentation Ethics Committee. All experimental procedures including the care, handling and maintenance of the experimental animals were performed according to the National Health and Medical Research Council guidelines for the use and care of animals for experimental purposes.

An NO-deficient rat model of hypertension was used. This animal model involves the inhibition of endogenous NO production with *N* ^G^-nitro-l-arginine methyl ester (l-NAME) that leads to an elevation in BP^(^ ^11^ ^)^. In this experiment, 12-week-old male Sprague–Dawley rats (Animal Resources Centre, Canning Vale, WA, Australia) were fed a standard laboratory rat diet (Glen Forrest Stock Feeders, Glen Forrest, WA, Australia). OPP was provided as a drink (30 ml/rat per d) from the start of the experiment at two different concentrations: 1500 mg/l gallic acid equivalent (GAE) and 3000 mg/l GAE. After 4 weeks on OPP, rats were challenged with l-NAME (final dosage of 15 mg/kg), and the treatments were continued for a further 8 weeks. The four experimental groups included (1) control, (2) l-NAME, (3) l-NAME+OPP (1500 mg/l GAE) and (4) l-NAME+OPP (3000 mg/l GAE).

BP was monitored fortnightly by a standard photoelectric tail-cuff procedure (IITC Life Sciences, Woodlands Hills, CA, USA) as described previously^(^ ^12^ ^,^ ^13^ ^)^. Briefly, pre-trained rats were placed in restraining tubes (IITC models 805 and 815), and the tails were occluded with an appropriate-sized cuff coupled to a pneumatic pulse transducer and electrophysiograph (IITC model 65-12). The pulse was detected as the cuff pressure was reduced, and the pressure at which the first pulse was detected was taken as the systolic BP and computed automatically (IITC software package). The ambient temperature was carefully controlled (30°C), and the average of three to four readings was taken as the final reading.

### Cardiac arrhythmia studies

In this experiment, 4-week-old male Wistar Kyoto rats (*n* 25), sourced from the Animal Resources Centre, were fed a pro-arrhythmic diet (consisted of a standard laboratory diet further supplemented with 5 % (w/w) lard; Glen Forrest Stock Feeders) for 4 months in the presence or absence of OPP. OPP was provided as the drinking fluid (35 ml/rat per d) at a concentration of 1500 mg/l GAE. The control group received water. At the end of the feeding period, animals were subjected to acute myocardial ischaemia for 30 min by a temporary occlusion of the left anterior descending coronary artery, and the vulnerability to ventricular tachycardia, ventricular fibrillation (VF) and sudden cardiac death was quantified^(^ ^14^ ^,^ ^15^ ^)^.

### Atherosclerosis studies

A total of twenty-seven male New Zealand White rabbits, aged 4–5 months, were obtained from the Institute of Medical Research, Kuala Lumpur, Malaysia, after ethical clearance on all animal experimental procedures involving animals was obtained from the Animal Care and Use Committee of the National University of Malaysia, Bangi, Selangor, Malaysia. These animals were divided into three groups of nine and fed an atherogenic diet for 100 d. The diet contained 35 % energy from fat, 40 % energy from carbohydrate and 25 % energy from protein, with added cholesterol (0·15 %, w/w). The fat comprised 67·1 % SFA, primarily as lauric acid (12 : 0) and myristic acid (14 : 0). The control group was fed the atherogenic diet and provided distilled water as the sole drinking-water. OPP were tested at two concentrations in two groups of nine animals each, and these animals were fed the atherogenic diet and supplemented with OPP at a concentration of either 500 or 1000 mg/l GAE in the drinking fluid. At the end of the animal feeding, the rabbits were killed by exsanguination following anaesthetisation with a mixture of ketamine and zoletil (0·1 ml/kg body weight), and entire aortas were traced, dissected and fixed in 10 % (v/v) formalin. The aortas were then stained with Oil Red-O, which resulted in lipid deposits in the aortas being stained red. Delineation of atheromatous deposits in the intima and lesions were quantified using a digital image analysis system, as described by Paigen *et al.* ^(^ ^16^ ^)^. Fibrous and fatty plaques, fatty streaks and lesion-free areas of the aorta were quantified as percentage of total aorta area examined.

### Anti-diabetic studies and clinical biochemistry analysis

To examine OPP for possible protective effects against diabetes, Nile rats (*Arvicanthis niloticus*), also known as African grass rats, from the Brandeis University breeding colony, were studied as the model of choice. In captivity, Nile rats fed a standard rat chow develop type 2 diabetes and exhibit all aspects of the metabolic syndrome examined to date^(^ ^17^ ^,^ ^18^ ^)^. All animal experiments were conducted in accordance with protocols approved by the Animal Care Committee of Brandeis University.

A total of fourteen 12-week-old male Nile rats fed a standard rat chow (Lab Diet no. 5020; Purina Mills, St Louis, MO, USA) were divided into two equal groups and studied for a 17-week period. The treatment group drank OPP at 1800 mg/l GAE as their water supply, whereas the untreated group received distilled water. A shorter pilot study indicated that treatment effects could be expected between 900 and 1800 mg/l GAE. The animals were weighed initially and before killing by exsanguination under anaesthesia at the end of the 17-week period.

Fasting blood glucose was measured in tail blood following light anaesthesia (CO_2_+O_2_, 1:1 mixture) at study origin after the rats were deprived of food for 15 h overnight. Glucose was measured with an Elite XL glucometer (Bayer Company, Elkhart, IN, USA) standardised against a conventional spectrophotometric assay.

For terminal measurement of plasma TAG, total cholesterol (TC) and insulin, fasting blood samples were collected by cardiac puncture under O_2_/CO_2_ anaesthesia and placed in plastic vials moistened with EDTA. Plasma TAG and TC were determined spectrophotometrically using Infinity™ kits (TAG ref no. TR22421, TC ref no. TR13421; ThermoScientific, Waltham, MA, USA). Insulin was determined using an ELISA kit for rat insulin (Linco Research, St Charles, MO, USA).

Liver lipids (TAG and TC) were extracted from 0·1 g of tissue ground with 4 g of sodium sulphate using a 2:1 chloroform–methanol solution. Total extract was combined and dried under N_2_ and redissolved in 1 ml of chloroform. An aliquot (10–20 μl) of each sample was dried under N_2_ and dissolved in 50 μl of Triton X-100 and chloroform (1:1 by volume). The solution was dried extensively to remove chloroform. TAG and TC were then determined using the appropriate Infinity™ kits.

### Anti-tumour studies and microarray gene expression analysis

In this experiment, 6-week-old male inbred BALB/c mice were purchased from the Institute of Medical Research, after ethical clearance on all experimental procedures involving animals was obtained from the Animal Care and Use Committee of the University of Malaya, Kuala Lumpur, Malaysia. The mice were injected subcutaneously at the dorsum of the neck with IgA-secreting J558 mouse myeloma cells (ATCC TIB-6) after acclimatisation for 1 week. The mice were then divided into two groups, one group was given distilled water (*n* 15) and the other was given OPP (1500 mg/l GAE) (*n* 15). The mice were killed by exsanguination under anaesthesia after 4 weeks, when the presence of tumours was observed through palpation at the neck. Tumours were excised, weighed, their dimensions (length, width and height) measured, snap-frozen in liquid N_2_ and stored at − 80°C. Tumour volume was calculated with the formula π/6 × length × width × height, which has the highest correlation with tumour mass as this is the formula of an ellipsoid^(^ ^19^ ^,^ ^20^ ^)^, which was the shape of the subcutaneous J558 tumours in the present study.

All precautions in handling RNA were taken in the present study. Total RNA isolation from mouse tumours was carried out using the RNeasy Mini Kit (Qiagen, Inc., Valencia, CA, USA) and QIAshredder homogeniser (Qiagen, Inc.). The total RNA samples obtained were subjected to a NanoDrop 1000A Spectrophotometer for yield and purity assessment. Integrity of the total RNA samples was then assessed using the Agilent 2100 Bioanalyser (Agilent Technologies, Santa Clara, CA, USA) and Agilent RNA 6000 Nano Chip Assay Kit (Agilent Technologies). A total of four total RNA samples with the highest RNA integrity numbers and 28S/18S rRNA ratios within each condition were then selected for microarray studies.

Amplification of total RNA samples, which were of high yield, purity and integrity, was carried out using the Illumina TotalPrep RNA Amplification Kit (Ambion, Inc., Austin, TX, USA). The biotinylated-complementary RNA produced was then hybridised to the Illumina MouseRef-8 Expression BeadChip version 1 (Illumina, Inc., San Diego, CA, USA) using the Direct Hybridisation Kit (Illumina, Inc.). Illumina MouseRef-8 Expression BeadChips contained 50-mer gene-specific probes for over 24 000 genes, which were designed based on the Mouse Exonic-Evidence-Based Oligonucleotide set, the RIKEN FANTOM 2 database and the National Center for Biotechnology Information RefSeq (Release 5) transcript database. Microarray hybridisation, washing and scanning were carried out according to the manufacturer's instructions.

In brief, complementary RNA was added to a hybridisation buffer, and the hybridisation mixture was then briefly heated and hybridised to an Illumina BeadChip. The hybridised microarray then underwent a series of washes using the wash buffers provided and 100 % (v/v) ethanol (Merck, Darmstadt, Germany). Non-specific hybridisation was blocked before incubating the microarray with the Amersham Fluorolink Streptavidin Cy-3 dye (GE Healthcare Bio-Sciences, Little Chalfont, UK) for detection, followed by a final wash with the wash buffer. The microarray was then dried and scanned with the Illumina BeadArray Reader confocal scanner and Illumina BeadScan software (Illumina, Inc.), available at the Malaysia Genome Institute, National University of Malaysia. The raw gene expression data obtained are available at Gene Expression Omnibus^(^ ^21^ ^)^ (accession no. GSE17614).

Quality control of the hybridisation, microarray data extraction and initial analysis were carried out using the Illumina BeadStudio software (Illumina, Inc.). Outlier samples were removed via hierarchical clustering analysis provided by the Illumina BeadStudio software and also using the TIGR MeV software (Institute for Genomic Research, Rockville, MD, USA)^(^ ^22^ ^)^, via different distance metrics. A minimum of three replicates per condition (with outliers removed) was then considered for further analysis. Gene expression values were normalised using the rank invariant method, and genes that had a detection level of more than 0·99 in either the control or treatment samples were considered significantly detected. To filter the data for genes that changed significantly in terms of statistics, the Illumina custom error model was used and genes were considered significantly changed at a ‘Differential Score’ of more than 13, which was equivalent to a *P* value of < 0·05.

The genes and their corresponding data were then exported into the Microsoft Excel software (Microsoft Corporation, Richmond, WA, USA) for further analysis. To calculate fold changes, an arbitrary value of 10 was given to expression values, which were less than 10. Fold changes were then calculated by dividing means of Signal Y (treatment) with means of Signal X (control) if the genes were up-regulated and vice versa if the genes were down-regulated. A two-way (gene and sample) hierarchical clustering of the significant genes was then performed using the TIGR MeV software^(^ ^22^ ^)^ to ensure that the replicates of each condition were clustered to each other. The Euclidean distance metric and average linkage method were used to carry out the hierarchical clustering analysis.

Changes in biological pathways and gene ontologies were assessed via functional analysis, using GenMAPP^(^ ^23^ ^)^ and MAPPFinder^(^ ^24^ ^)^ software (both University of California at San Francisco, San Francisco, CA, USA). The MAPPFinder software ranks GenMAPP (pathways) and gene ontologies based on the hypergeometric distribution. Readers are referred to Doniger *et al.* ^(^ ^24^ ^)^ for further explanations of the terms used in the MAPPFinder software. GenMAPP and gene ontologies that had permuted *P* values of less than 0·05, number of genes changed of more than or equal to 2 and *Z* scores of more than 2 were considered significant. Boxes coloured yellow indicate genes that were up-regulated, while those coloured blue indicate genes that were down-regulated. Individual boxes that have different shadings within them indicate the presence of multiple probes (splice transcripts) within a single gene.

Changes in regulatory networks were also analysed through the use of the Ingenuity Pathways Analysis software (Ingenuity Systems, Redwood City, CA, USA). For each organ, a dataset containing differentially expressed genes and their corresponding fold changes was uploaded into the application. Analysis of up-regulated and down-regulated genes was carried out separately. Each gene identifier was mapped to its corresponding gene object in the Ingenuity Pathways Knowledge Base. These genes were then overlaid onto a global molecular network developed from information contained in the Ingenuity Pathways Knowledge Base. Networks of these focus genes were then algorithmically generated based on their connectivity. A network is a graphical representation of the molecular relationships between genes or gene products. Genes or gene products were represented as nodes (shapes), and the biological relationship between two nodes was represented as an edge (line). The intensity of the node colour indicates the degree of up-regulation (red) or down-regulation (green). Nodes were displayed using various shapes that represented the functional class of the gene product. Edges were displayed with various labels that described the nature of the relationship between the nodes.

We carried out two-step real-time quantitative RT-PCR (qRT-PCR) studies on target genes selected to represent the tumours used in the microarray experiments, using TaqMan Gene Expression Assays (Applied Biosystems, Foster City, CA, USA). Primer and probe sets for the selected genes were obtained from the ABI Inventoried Assays-On-Demand (Applied Biosystems). Reactions were carried out according to the manufacturer's instructions. Quality control of the replicates used, real-time qRT-PCR data extraction and initial analysis were carried out using the 7000 Sequence Detection System software (Applied Biosystems). Relative quantification of the target genes of interest was carried out using the qBase 1.3.5 software (Center for Medical Genetics, Ghent University Hospital, Ghent, Belgium)^(^ ^25^ ^)^, which takes into account the calculations of amplification efficiencies and multiple reference genes. Expression levels of target genes were normalised to the geometric mean of the three most stable reference genes, chosen from the four tested, namely RAS p21 protein activator 1 (*Rasa1*), sema domain Ig domain (Ig) transmembrane domain and short cytoplasmic domain (semaphorin) 4A (*Sema4a*), protein-tyrosine sulfotransferase 2 (*Tpst2*), and eukaryotic 18S rRNA endogenous control (18S rRNA). The first three reference genes were selected based on the microarray data, while the fourth is a common reference gene used in real-time qRT-PCR experiments. Stability of these reference genes was assessed using the geNorm 3.5 software (Center for Medical Genetics)^(^ ^26^ ^)^.

### Statistical analysis

Data were analysed using the Statistical Analysis System program. The experimental results are expressed as means and standard deviations, unless otherwise stated. A two-tailed unpaired Student's *t* test (comparison of two groups) or ANOVA (comparison of more than two groups) was performed, and significant differences between means were determined. Where appropriate, the required *post hoc* statistical analyses were applied for a more robust outcome. These are indicated and explained in the appropriate tables and figures. For all outcomes, *P*< 0·05 was considered statistically significant.

## Results and discussion

### Oil palm phenolic samples

OPP in oil palm vegetation liquor ranged from 1·38 to 2·43 % on a dry weight basis^(^ ^10^ ^)^. The remaining 98 % on a dry weight basis consists of fruit sugars, soluble fibres, organic acids and water-soluble vitamins, etc. It is known that phenolics are minor components in all plants, similar to other phytonutrients such as carotenoids and tocols (vitamin E). However, they are only needed in small quantities to affect a biological response. Since the present study is the first to test the effects of OPP on animals, we arbitrarily chose 500 mg/l GAE as the lowest concentration to investigate whether there were any biological effects on animals. Having determined the positive effects, we moved on to test a whole range of concentrations, with 500 mg/l GAE as the lowest dose and 3000 mg/l GAE as the highest dose. For the anti-diabetic study with Nile rats, a shorter pilot study has indicated that treatment effects could be expected between 900 and 1800 mg/l GAE (data not shown). As such, in the present study, we report the results for the highest dose tested (1800 mg/l GAE). In rat studies, the volume of OPP given was 30–35 ml/d for all concentrations tested. Based on an average rat weight of 300 g, this would be equivalent to the consumption of about 1000 ml of OPP by a 60 kg human subject, which is calculated based on the body surface area normalisation method^(^ ^27^ ^)^.

### Cardiovascular protection

*In vivo* animal studies assessed the possible cardioprotective effects of OPP. These include studies on BP regulation in rats treated with l-NAME, an inhibitor of NO synthase, cardiac arrhythmia studies using rat models of coronary artery ligation and atherosclerosis studies using rabbits.

**Fig. 1:**
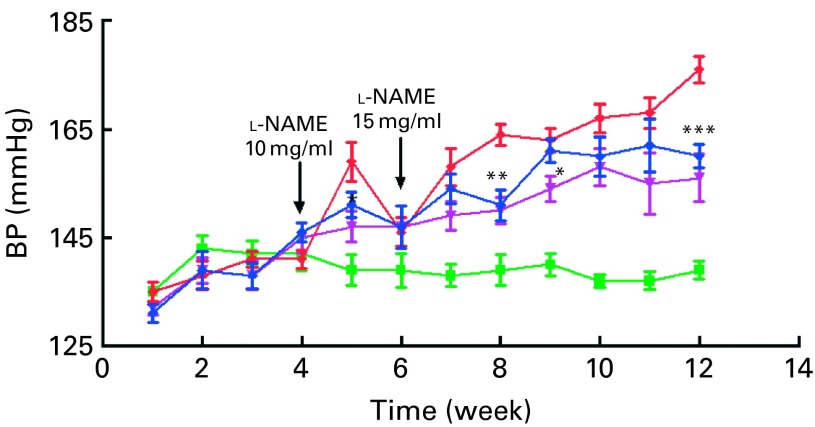
Reduction in blood pressure (BP) by oil palm phenolics (OPP). Values were significantly different as assessed by two-tailed unpaired Student's *t* test compared with the *N* ^G^-nitro-l-arginine methyl ester (l-NAME) group (*n* 12): * *P*< 0·05, ** *P*< 0·01, *** *P*< 0·001. 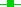, Control; 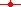, l-NAME; 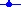, l-NAME+OPP (1500 gallic acid equivalents (GAE)); 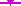, l-NAME+OPP (3000 GAE).

In BP regulation studies, 12-week-old Sprague–Dawley rats were supplemented at two different doses of OPP (1500 and 3000 mg/l GAE) for 4 weeks before being treated with l-NAME. At 10 mg/kg, l-NAME resulted in a sharp rise in BP (18 mmHg) in the l-NAME control group (Fig. 1). However, no such initial rise was observed in rats that were treated with OPP. An increase in l-NAME dosage to 15 mg/kg resulted in a gradual increase in BP in OPP-treated rats over time. This was in contrast to the untreated control group, where l-NAME-treated (15 mg/kg) rats developed significantly higher BP values. This protection against hypertension by OPP was greater in rats at the higher dose (Fig. 1).

Oral administration of OPP thus lowered BP in a NO-deficient rat model of hypertension and is consistent with the observations made with isolated vascular preparations described in the previous study^(^ ^8^ ^)^. In addition to the possible direct effect on the endothelial NO synthase system, OPP being an antioxidant may have scavenged the increased reactive oxygen species associated with l-NAME, thus reducing or inhibiting oxidative stress central to the development of hypertension and atherosclerosis. Therefore, OPP may have broad applications in modulating NO in chronic syndromes such as CVD.

The potential cardioprotective effect of OPP was also evaluated using an *in vivo* rat model of cardiac arrhythmia and sudden cardiac death, following a long-term dietary regimen. At the end of the 4-month feeding period, rats were subjected to myocardial ischaemia by ligating and hence occluding the coronary artery. The vulnerability to ventricular tachycardia, VF and sudden cardiac death was measured. The incidence of ventricular tachycardia was not influenced by OPP, but there was a trend (not significant) towards a lower mean duration of ventricular tachycardia (Table 1). In contrast, OPP at 1500 mg/l in drinking-water resulted in a significantly lower incidence (*P*< 0·05, χ^2^) of VF. Of the twenty-five rats, thirteen (52 %) went into VF compared with twenty-one out of twenty-three rats (91 %) in the unsupplemented controls. Rats receiving OPP also had a significantly shortened (*P*< 0·05 or better) duration of VF. The overall mortality in the OPP group was five out of twenty-five (20 %) compared with nine out of twenty-three (40 %) in the control group. No significant difference was evident in the area of the myocardium that was rendered ischaemic by ligation of the coronary artery, suggesting that these observations were independent of the surgical procedure.

**Table 1:** Reduction of vulnerability to ventricular tachycardia (VT), ventricular fibrillation (VF) and mortality in rat models of cardiac arrhythmia (Mean values, standard deviations and percentages)

	Distilled water (control) (*n* 23)	OPP (1500 mg/l GAE) (*n* 25)
Parameters measured	Mean	sd	Mean	sd
VT incidence (% of animals)	96	88
VT duration (s)	120·5	35·9	85·2	26·1
VF incidence (% of animals)	91	52*
VF duration (s)	99·8	21·2	47·5*	15·5
Mortality (% of animals)	40	20
Ischaemic myocardium area zone-at-risk (%)	49·6	0·7	48·6	0·8

OPP, oil palm phenolics; GAE, gallic acid equivalent.

* Values were significantly different (*P*< 0·05).

As atherosclerosis represents an important component of CVD, the development of atherosclerosis was assessed in rabbits fed a high-fat, cholesterol-rich atherogenic diet. As expected, control rabbits fed this atherogenic diet developed extensive fibrous and fatty plaques as well as fatty streaks in their aortas (Table 2). On the other hand, animals given the same atherogenic diet but supplemented with OPP as drinking fluid resulted in significantly reduced fatty plaques and fatty streaks. In addition, lesion-free areas in OPP-supplemented animals were more prevalent, indicating a net protection against the development and progression of atherosclerosis. The morphology and progression of atheromatous lesions in rabbits were similar to that described in human atheromatous lesions^(^ ^28^ ^)^. While the results obtained may not be directly translated to human subjects, it is nonetheless strong evidence of bioactivity and hence bioavailability.

**Table 2:** Inhibition of the development of aortic lesions in rabbits fed an atherogenic diet* (Mean values and standard deviations, *n* 9)

	Distilled water (control)	OPP (500 mg/l GAE)	OPP (1000 mg/l GAE)
Description of lesion	Mean	sd	Mean	sd	Mean	sd
Fibrous plaque†	7·96^a,b^	3·82	1·41^a,c^	0·81	0·96^b,c^	0·45
Fatty plaque‡	8·53^a,b^	3·47	4·82^a^	2·66	3·49^b^	2·98
Fatty streak§	12·41^a,b^	5·02	10·28^a,c^	5·74	6·45^b,c^	3·72
Lesion-free area∥	70·93^a,b^	6·76	83·40^a^	8·04	87·92^b^	8·12
Total area (%)	99·83	0·28	99·97	0·16	99·85	0·27

OPP, oil palm phenolics; GAE, gallic acid equivalent.

^a,b,c^Mean values with like superscript letters were significantly different from each other (*P*< 0·05).

* Values are percentage of total aorta area (mm^2^).

† Raised nodular lesions, continuous, intense red, white hard and visible to naked eyes.

‡ Raised distinct lesions, intensely stained red.

§ Lipid accumulation, stained light red.

∥ Healthy intima.

### Protection against diabetes

Providing OPP *ad libitum* at 1800 mg/l GAE as the sole drinking fluid for 17 weeks blocked diabetes progression (hyperglycaemia and hyperlipaemia) in 12-week-old male Nile rats as evidenced by normalisation of initially elevated blood glucose and plasma lipids (Table 3). Rats that were not supplemented with OPP eventually progressed to severe polyuria and polydipsia with enlarged kidneys and livers. Fasting blood glucose levels at the start of the experiment indicated early diabetes in the OPP-treated group (1250 mg/l on average), which subsequently improved after 17 weeks of OPP treatment (480 mg/l), while glucose in the untreated rats rose from pre-diabetic (870 mg/l) to diabetic (1370 mg/l) levels. Normal glucose in Nile rats ranges from about 400 mg/l at weaning to about 900 mg/l as non-diabetic young adults. Values exceeding 4000 mg/l are common in non-fasting diabetic adult rats. Slightly higher plasma insulin in the treatment group suggests that OPP may have enhanced insulin production, even as β-cells failed in the controls^(^ ^29^ ^)^. Plasma lipids including TAG and TC in untreated rats were severely elevated by the end of the study compared with rats receiving OPP, which had plasma lipid values that were essentially normal.

**Table 3:** Anti-diabetic effects of oil palm phenolics (OPP)* (Mean values and standard deviations, *n* 7)

	Water	OPP (1800 mg/l GAE)
Parameter	Mean	sd	Mean	sd
Diet (kJ/g)	15·69		15·69	
Body weight (g)				
Initial (11–13 weeks old)	95	20	98	12
Final (at 30 weeks)	111	18	118	20
Body-weight gain (g/d)	0·16	0·21	0·29	0·24
Food intake (overall average)				
g/d	18†	5	12	3
kJ/d	276†	75	184	42
kJ/d per kg body weight	2548†	812	1594	423
Water or OPP intake				
At third month (ml/d)	66†	34	40	21
At third month (kJ/d)	0		46	25
Total energy intake				
Food+OPP (kJ/d)	276	75	230	42
OPP intake at third month (mg/d per kg body weight)	0		508	
Organ weight (% body weight)				
Liver	5·4†	1·4	3·8	0·6
Kidney	1·4†	0·7	0·9	0·2
Adipose (total)	6·3	1·0	8·3	3·0
Carcass	68	2	70	1
Fasting blood glucose (mg/l)				
Initial	870	960	1250	1500
Final (at 16–17 weeks)	1390†	1090	480	160
Final plasma insulin (ng/ml)	3·38	1·57	6·20	2·43
Plasma TAG (mg/l)				
Initial	850	350	990	1020
Final (at 17 weeks)	2240†	1890	650	300
Plasma TC (mg/l)				
Final (at 17 weeks)	4010†	2380	1720	780

GAE, gallic acid equivalent; TC, total cholesterol.

* Male Nile rats, 12 weeks old, were fed standard chow 5020 and drinking-water or OPP for 17 weeks.

† Mean values were significantly different (*P*< 0·05).

Wild-type Nile rats exhibit extensive variation in their rate of developing diabetes^(^ ^17^ ^)^, so the plasma TC and TAG elevation varies based on their degree of diabetes. But when the extent of individual hyperglycaemia is correlated with TC, and especially with TAG, the relationship is extremely powerful, with *R* correlation values obtained typically >0·70–0·75. This is also true for relationships between liver weight and blood glucose, and between kidney weight and blood glucose. Thus, the parameters evaluated in the present study (glucose, TC, TAG, liver and kidney weights) are good markers of diabetic status and the metabolic syndrome, and all are highly interrelated, as one would predict for diabetes and the metabolic syndrome in human subjects. Elevated TAG, hyperglycaemia, depressed HDL and fatty liver are common to this animal model and the human disease^(^ ^17^ ^)^. It is important to note that while some of the seven control rats were diabetic by the end of the present study (blood glucose >1100 mg/l), none of the OPP group was diabetic (blood glucose < 1000 mg/l). The probability that this was not due to chance was significant at *P*< 0·05, with the means being especially supported by the observation that blood glucose levels in most of the rats receiving OPP declined, whereas those in most of the controls increased. Expressing the data as percentage of change would have been more striking, but that would deprive the reader of appreciating the true biological variation that was observed in the present study. Furthermore, the effect of OPP on diabetes was confirmed statistically (despite the inherent variation) by all the other criteria (TAG, TC, liver and kidney weights).

The present results thus imply that OPP protects against (and may even reverse) early type 2 diabetes characterised by hyperglycaemia and hyperlipaemia in a model that develops spontaneous diabetes when fed typical rat chow. This is in line with the knowledge that phenolics confer anti-diabetic effects via various mechanisms, including reducing oxidative stress^(^ ^30^ ^)^ and lipid peroxidation^(^ ^31^ ^)^, defending pancreatic cells from oxidative damage^(^ ^32^ ^)^, protecting lymphocytes from DNA damage^(^ ^33^ ^)^, as well as preserving vascular functions in diabetic complications^(^ ^34^ ^)^. Mulvihill *et al.* ^(^ ^35^ ^)^ reported that naringenin attenuated dyslipaemia and hyperinsulinaemia in LDL receptor null mice with diet-induced insulin resistance. Anderson^(^ ^36^ ^)^ reported that cinnamon polyphenols improved insulin sensitivity *in vitro* and in individuals with type 2 diabetes. Subjects with the metabolic syndrome who consumed an aqueous extract of cinnamon have been shown to have improved fasting blood glucose, systolic BP, percentage body fat and lean body mass compared with the placebo group. Huang *et al.* ^(^ ^37^ ^)^ investigated the effects of caffeic and cinnamic acids on glucose uptake by insulin-resistant mouse liver FL83B cells. Their results suggested that these phenolic acids promoted insulin receptor tyrosyl phosphorylation, up-regulated the expression of insulin signal-associated proteins (insulin receptor, phosphatidylinositol-3 kinase, glycogen synthase and GLUT-2), increased the uptake of glucose, and therefore alleviated insulin resistance in the cells.

Insulin resistance is the main trigger for the metabolic syndrome and is a consequence of the inability of insulin to efficiently signal its receptor kinase and/or downstream targets. Assuming OPP can be found to attenuate metabolic disorders linked to insulin resistance in other systems, it holds promise for this aspect of the metabolic syndrome.

### Anti-tumour effects

**Fig. 2:**
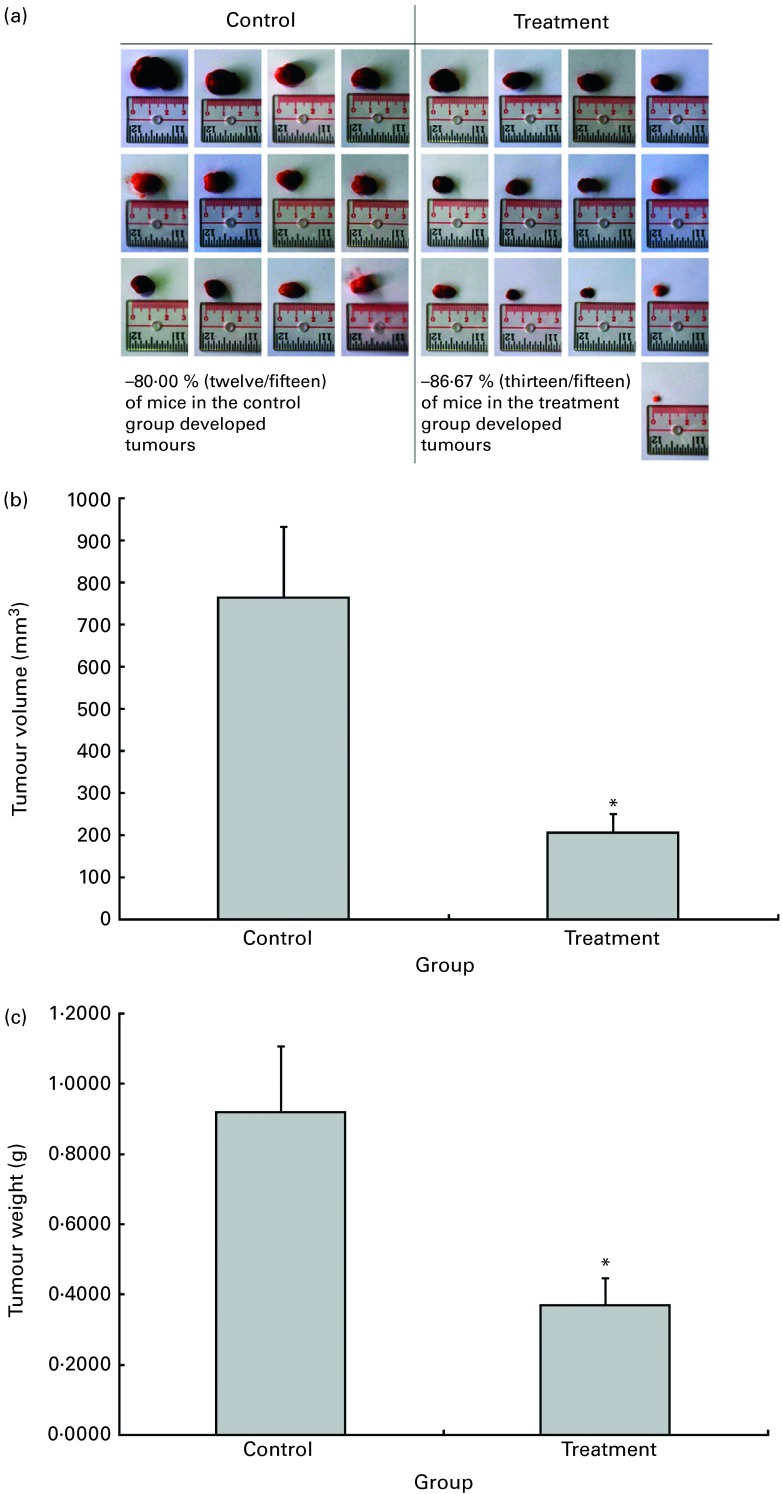
*In vivo* anti-tumour effects of oil palm phenolics. (a) Tumour incidence, (b) tumour volume reduction and (c) tumour weight reduction. * Values were significantly different as assessed by the two-tailed unpaired Student's *t* test, *n* 12–13 (*P*< 0·05).

The possible anti-tumour properties of OPP were studied using 7-week-old BALB/c mice subcutaneously inoculated with syngeneic J558 mouse myeloma cells. All animals were given standard rodent chow in this experiment. The test animals were given OPP (1500 mg/l GAE) *ad libitum* as the sole fluid source, while the control animals received distilled water. No significant differences were observed in tumour incidence (Fig. 2(a)), as the entire tumour mass typically grows at the site of injection. However, this experiment revealed a significant reduction in tumour volume (Fig. 2(b)) and weight (Fig. 2(c)) in the OPP-treated group.

**Fig. 3:**
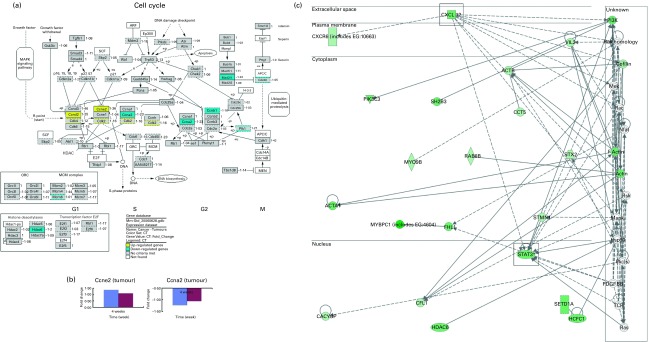
Microarray analysis results obtained from the anti-tumour studies of oil palm phenolics (OPP). (a) Genes differentially expressed in the tumour cell cycle pathway as indicated by the GenMAPP analysis indicate cytostatic effects of OPP (Illumina custom error model *P*< 0·05, *n* 3–4). The figure is adapted from KEGG and maintained by GenMAPP.org. (b) Gene expression fold changes of two cell-cycle genes as determined by microarray and real-time quantitative RT-PCR experiments were comparable, thus validating the microarray results obtained. (c) Genes down-regulated in the *Stat3* and *Cxcl12* network generated by the Ingenuity Pathways Analysis software indicate possible anti-invasiveness and anti-metastatic potential of OPP. 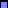, Microarray; 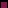, real-time quantitative RT-PCR. ORC, origin recognition complex; MCM, mini-chromosome maintenance; MAPK, mitogen-activated protein kinase; SCF, *Skp1*/*Cul1*/F-box complex; ARF, alternative reading frame; APC/C, anaphase-promoting complex/cyclosome; MEN, mitotic exit network; CT, cancer-tumours.

As an extension to this anti-tumour observation, we explored the possible molecular mechanisms involved by using microarray analysis. Functional analysis carried out using microarray data obtained from tumours of mice indicated that genes involved in the cell cycle were differentially expressed (Fig. 3(a)) in the OPP-treated group. The genes and functions significantly affected by OPP are listed in supplementary materials (available online at http://www.journals.cambridge.org/bjn). On the one hand, genes such as cyclin D2 (*Ccnd2*), cyclin E2 (*Ccne2*), cyclin-dependent kinase 4 (*Cdk4*) and cyclin-dependent kinase 2 (*Cdk2*) were up-regulated by OPP. On the other hand, genes such as cyclin A2 (*Ccna2*), cyclin B1 (*Ccnb1*), polo-like kinase 1 (*Plk1*), mitotic arrest deficient 2, homologue-like 1 (*Mad2l1*), cell division cycle 20 homologue (*Cdc20*), histone deacetylase 6 (*Hdac6*) and minichromosome maintenance deficient 6 (*Mcm6*) were down-regulated by OPP. The directions and fold changes for two of these genes, *Ccne2* and *Ccna2*, obtained from real-time qRT-PCR, were comparable with those obtained using the microarray technique (Fig. 3(b)), thus validating the microarray results obtained.

The regulation of cell-cycle genes found in the present study suggests that OPP may inhibit tumour growth *in vivo* by inducing a G1/S phase arrest in the cell cycle^(^ ^38^ ^)^. This is consistent with observations made in several cancer cell lines, in which cell-cycle arrest was brought about by other phenolic antioxidants such as phenolic acids^(^ ^39^ ^)^, hydroxytyrosol^(^ ^40^ ^,^ ^41^ ^)^, resveratrol^(^ ^42^ ^)^, piceatannol^(^ ^43^ ^)^, genistein^(^ ^44^ ^)^ and luteolin^(^ ^45^ ^)^. Interestingly, the same genes that we found to be significantly changed by OPP were also found to be regulated, but in the opposite direction, in colorectal tumour cells resistant to a combined chemotherapy of folinic acid, 5-fluorouracil and irinotecan^(^ ^46^ ^)^. The opposite direction of regulation of these genes by OPP thus indicates that OPP may help sensitise tumour cells towards chemotherapy, and suggests that it might be used together with selected chemotherapeutic agents for enhanced cytotoxicity in cancer cells.

Furthermore, genes that are normally associated with cancer proliferation and invasion such as signal transducer and activator of transcription 3 (*Stat3*) and chemokine (C–X–C motif) ligand 12 (*Cxcl12*) were also down-regulated by OPP (Fig. 3(c)). *Stat3* signalling is oncogenic and is required for the transformation of various primary cancer and tumour-derived cell lines. Thus, many cancer-derived cell lines that contain constitutively activated *Stat3* are dependent on this protein, and they undergo growth arrest or apoptosis when treated with antisense or dominant negative constructs directed at *Stat3* ^(^ ^47^ ^)^. Niu *et al.* ^(^ ^48^ ^)^ showed that blocking *Stat3* in cancer cells up-regulates the expression of *p*53, leading to *p*53-mediated tumour cell apoptosis. In addition, inhibition of constitutive *Stat3* activity in haematopoietic malignancies such as non-Hodgkin's lymphoma and multiple myeloma sensitises these resistant cancers to chemotherapeutic drug-mediated apoptosis^(^ ^49^ ^)^, as well as suppresses the growth of prostate^(^ ^50^ ^)^ and astrocytoma^(^ ^51^ ^)^ cancer cells. In agreement with this, resveratrol, which has been reported to have anti-tumour properties, was also found to inhibit *Stat3* signalling in transformed mouse fibroblasts as well as human breast, pancreatic and prostate carcinoma cell lines^(^ ^52^ ^)^.

The most invasive cancers normally produce the broadest spectrum and the highest levels of chemokines^(^ ^53^ ^)^. An important example of a cytokine receptor pair involved in cancer invasion and metastasis is *Cxcl12*/*Cxcr4*. *In vitro*, the CXCL12 ligand stimulated breast cancer cells to carry out the basics of invasion, including pseudopodial protrusion, directed migration and penetration of extracellular matrix barriers^(^ ^54^ ^)^. *In vivo*, metastasis to CXCL12-rich lung tissue was blocked in animal models by treatment with a neutralising anti-human CXCR4 monoclonal antibody^(^ ^54^ ^)^. *Cxcl12* is also an important mediator of advanced cancer and contributes significantly to the lethal phenotype^(^ ^55^ ^)^. Tannic acid, a water-soluble polyphenol, which is abundant in Chinese herbal medicines and tea, is a *Cxcl12*/*Cxcr4* inhibitor, and this activity may contribute to its anti-inflammatory, anti-angiogenic and anti-tumour properties^(^ ^56^ ^)^. In the present study, the down-regulation of *Cxcl12* in tumours of OPP-treated mice thus suggests a reduction in invasiveness and, possibly, metastatic potential of these tumours.

### Conclusions

The array of bioactivities displayed by OPP suggests its potential application for a range of chronic diseases. While the biological effects observed in the present study are mainly attributed to phenolic compounds, the possible effects of other components cannot be discounted. What is important is that the extract in its entirety confers the positive outcomes reported in the present study. However, the effects of OPP in human subjects have yet to be shown, and we are currently designing human intervention trials to address this issue. Although we do not have information on the actual bioactive metabolites that reach the target organs, the breadth of biological activities observed in the present study indicates that OPP is bioavailable in animals and triggers a central regulatory response, which modulates key metabolic functions. In addition to the anti-tumour mechanisms observed in the present study, the use of genome-scale techniques to identify the molecular modes of action involved in conferring the biological effects of OPP in other chronic diseases is thus very much called for.

## References

[1] Duttaroy AK & Jorgensen A (2004) Effects of kiwi fruit consumption on platelet aggregation and plasma lipids in healthy human volunteers. Platelets 15, 287–292 15370099 10.1080/09537100410001710290

[2] Gorinstein S, Caspi A, Libman I, et al. (2004) Preventive effects of diets supplemented with sweetie fruits in hypercholesterolemic patients suffering from coronary artery disease. Prev Med 38, 841–847 15193907 10.1016/j.ypmed.2003.12.021

[3] Decker EA (1995) The role of phenolics, conjugated linoleic acid, carnosine, and pyrroloquinoline quinone as nonessential dietary antioxidants. Nutr Rev 53, 49–58 7770184 10.1111/j.1753-4887.1995.tb01502.x

[4] Kuiper GG, Lemmen JG, Carlsson B, et al. (1998) Interaction of estrogenic chemicals and phytoestrogens with estrogen receptor beta. Endocrinology 139, 4252–4263 9751507 10.1210/endo.139.10.6216

[5] Grassi D, Lippi C, Necozione S, et al. (2005) Short-term administration of dark chocolate is followed by a significant increase in insulin sensitivity and a decrease in blood pressure in healthy persons. Am J Clin Nutr 81, 611–614 15755830 10.1093/ajcn/81.3.611

[6] Bhushan S, Kalia K, Sharma M, et al. (2008) Processing of apple pomace for bioactive molecules. Crit Rev Biotechnol 28, 285–296 19051107 10.1080/07388550802368895

[7] Cao X, Wang C, Pei H, et al. (2009) Separation and identification of polyphenols in apple pomace by high-speed counter-current chromatography and high-performance liquid chromatography coupled with mass spectrometry. J Chromatogr A 1216, 4268–4274 19203755 10.1016/j.chroma.2009.01.046

[8] Sambanthamurthi R, Tan YA, Sundram K, et al. (2011) Oil palm vegetation liquor: a new source of phenolic bioactives. Br J Nutr 106, 1655–1663 21736792 10.1017/S0007114511002121 PMC4179495

[9] Sambanthamurthi R, Tan YA, Sundram K (2008) Treatment of vegetation liquors derived from oil-bearing fruit. United States Patent US 7,387,802 B2. Malaysian Palm Oil Board

[10] Sambandan TG, Rha CK, Sinskey AJ, et al. (2010) Composition comprising caffeoylshikimic acids, protocatechuic acid, hydroxytyrosol, hydroxybenzoic acid and their derivatives and method of preparation thereof. World Patent WO 2010/137943. Malaysian Palm Oil Board

[11] Bernatova I, Pechanova O, Babal P, et al. (2002) Wine polyphenols improve cardiovascular remodeling and vascular function in NO-deficient hypertension. Am J Physiol Heart Circ Physiol 282, H942–H948 11834490 10.1152/ajpheart.00724.2001

[12] Mano MT, Bexis S, Abeywardena MY, et al. (1995) Fish oils modulate blood pressure and vascular contractility in the rat and vascular contractility in the primate. Blood Press 4, 177–186 7670652 10.3109/08037059509077591

[13] Chen Q, Gruber H, Swist E, et al. (2009) Influence of dietary phytosterols and phytostanols on diastolic blood pressure and the expression of blood pressure regulatory genes in SHRSP and WKY inbred rats. Br J Nutr 102, 93–101 19025722 10.1017/S0007114508137904

[14] McLennan PL, Abeywardena MY & Charnock JS (1988) Dietary fish oil prevents ventricular fibrillation following coronary artery occlusion and reperfusion. Am Heart J 116, 709–717 3414486 10.1016/0002-8703(88)90328-6

[15] Abeywardena MY & Charnock JS (1995) Dietary lipid modification of myocardial eicosanoids following ischemia and reperfusion in the rat. Lipids 30, 1151–1156 8614306 10.1007/BF02536617

[16] Paigen B, Morrow A, Holmes PA, et al. (1987) Quantitative assessment of atherosclerotic lesions in mice. Atherosclerosis 68, 231–240 3426656 10.1016/0021-9150(87)90202-4

[17] Chaabo F, Pronczuk A, Maslova E, et al. (2010) Nutritional correlates and dynamics of diabetes in the Nile rat (*Arvicanthis niloticus*): a novel model for diet-induced type 2 diabetes and the metabolic syndrome. Nutr Metab (Lond) 7, 29 20398338 10.1186/1743-7075-7-29 PMC2868017

[18] Noda K, Melhorn MI, Zandi S, et al. (2010) An animal model of spontaneous metabolic syndrome: Nile grass rat. FASEB J 24, 2443–2453 20335226 10.1096/fj.09-152678 PMC2887270

[19] Tomayko MM & Reynolds CP (1989) Determination of subcutaneous tumor size in athymic (nude) mice. Cancer Chemother Pharmacol 24, 148–154 2544306 10.1007/BF00300234

[20] Zielinski MR, Muenchow M, Wallig MA, et al. (2004) Exercise delays allogeneic tumor growth and reduces intratumoral inflammation and vascularization. J Appl Physiol 96, 2249–2256 15020578 10.1152/japplphysiol.01210.2003 PMC3645346

[21] Edgar R, Domrachev M & Lash AE (2002) Gene expression omnibus: NCBI gene expression and hybridization array data repository. Nucleic Acids Res 30, 207–210 11752295 10.1093/nar/30.1.207 PMC99122

[22] Saeed AI, Sharov V, White J, et al. (2003) TM4: a free, open-source system for microarray data management and analysis. Biotechniques 34, 374–378 12613259 10.2144/03342mt01

[23] Dahlquist KD, Salomonis N, Vranizan K, et al. (2002) GenmAPP, a new tool for viewing and analyzing microarray data on biological pathways. Nat Genet 31, 19–20 11984561 10.1038/ng0502-19

[24] Doniger SW, Salomonis N, Dahlquist KD, et al. (2003) MAPPFinder: using gene ontology and GenmAPP to create a global gene-expression profile from microarray data. Genome Biol 4, R7 12540299 10.1186/gb-2003-4-1-r7 PMC151291

[25] Hellemans J, Mortier G, De Paepe A, et al. (2007) qBase relative quantification framework and software for management and automated analysis of real-time quantitative PCR data. Genome Biol 8, R19 17291332 10.1186/gb-2007-8-2-r19 PMC1852402

[26] Vandesompele J, De Preter K, Pattyn F, et al. (2002) Accurate normalization of real-time quantitative RT-PCR data by geometric averaging of multiple internal control genes. Genome Biol 3, RESEARCH0034 10.1186/gb-2002-3-7-research0034 PMC126239 12184808

[27] Reagan-Shaw S, Nihal M & Ahmad N (2008) Dose translation from animal to human studies revisited. FASEB J 22, 659–661 17942826 10.1096/fj.07-9574LSF

[28] Yanni AE (2004) The laboratory rabbit: an animal model of atherosclerosis research. Lab Anim 38, 246–256 15207035 10.1258/002367704323133628

[29] Hayes KC, Sundram K & Sambanthamurthi R, et al. (2009) Methods for the treatment or prevention of diabetes mellitus and other metabolic imbalances. World Patent WO 2009/146102 A1. Brandeis University, Malaysian Palm Oil Board

[30] Fukuda T, Ito H & Yoshida T (2004) Effect of the walnut polyphenol fraction on oxidative stress in type 2 diabetes mice. Biofactors 21, 251–253 15630205 10.1002/biof.552210148

[31] Fenercioglu AK, Saler T, Genc E, et al. (2009) The effects of polyphenol-containing antioxidants on oxidative stress and lipid peroxidation in type 2 diabetes mellitus without complications. J Endocrinol Invest 33, 118–124 19834314 10.1007/BF03346565

[32] Lapidot T, Walker MD & Kanner J (2002) Antioxidant and prooxidant effects of phenolics on pancreatic beta-cells *in vitro* . J Agric Food Chem 50, 7220–7225 12452635 10.1021/jf020615a

[33] Lean ME, Noroozi M, Kelly I, et al. (1999) Dietary flavonols protect diabetic human lymphocytes against oxidative damage to DNA. Diabetes 48, 176–181 9892240 10.2337/diabetes.48.1.176

[34] Balzer J, Rassaf T, Heiss C, et al. (2008) Sustained benefits in vascular function through flavanol-containing cocoa in medicated diabetic patients a double-masked, randomized, controlled trial. J Am Coll Cardiol 51, 2141–2149 18510961 10.1016/j.jacc.2008.01.059

[35] Mulvihill EE, Allister EM, Sutherland BG, et al. (2009) Naringenin prevents dyslipidemia, apolipoprotein B overproduction, and hyperinsulinemia in LDL receptor-null mice with diet-induced insulin resistance. Diabetes 58, 2198–2210 19592617 10.2337/db09-0634 PMC2750228

[36] Anderson RA (2008) Chromium and polyphenols from cinnamon improve insulin sensitivity. Proc Nutr Soc 67, 48–53 18234131 10.1017/S0029665108006010

[37] Huang DW, Shen SC & Wu JS (2009) Effects of caffeic acid and cinnamic acid on glucose uptake in insulin-resistant mouse hepatocytes. J Agric Food Chem 57, 7687–7692 19685889 10.1021/jf901376x

[38] Schwartz GK & Shah MA (2005) Targeting the cell cycle: a new approach to cancer therapy. J Clin Oncol 23, 9408–9421 16361640 10.1200/JCO.2005.01.5594

[39] Kampa M, Alexaki VI, Notas G, et al. (2004) Antiproliferative and apoptotic effects of selective phenolic acids on T47D human breast cancer cells: potential mechanisms of action. Breast Cancer Res 6, R63–R74 14979919 10.1186/bcr752 PMC400651

[40] Fabiani R, De Bartolomeo A, Rosignoli P, et al. (2002) Cancer chemoprevention by hydroxytyrosol isolated from virgin olive oil through G1 cell cycle arrest and apoptosis. Eur J Cancer Prev 11, 351–358 12195161 10.1097/00008469-200208000-00006

[41] Fabiani R, De Bartolomeo A, Rosignoli P, et al. (2006) Virgin olive oil phenols inhibit proliferation of human promyelocytic leukemia cells (HL60) by inducing apoptosis and differentiation. J Nutr 136, 614–619 16484533 10.1093/jn/136.3.614

[42] Joe AK, Liu H, Suzui M, et al. (2002) Resveratrol induces growth inhibition, S-phase arrest, apoptosis, and changes in biomarker expression in several human cancer cell lines. Clin Cancer Res 8, 893–903 11895924

[43] Wolter F, Clausnitzer A, Akoglu B, et al. (2002) Piceatannol, a natural analog of resveratrol, inhibits progression through the S phase of the cell cycle in colorectal cancer cell lines. J Nutr 132, 298–302 11823594 10.1093/jn/132.2.298

[44] Raffoul JJ, Wang Y, Kucuk O, et al. (2006) Genistein inhibits radiation-induced activation of NF-kappaB in prostate cancer cells promoting apoptosis and G2/M cell cycle arrest. BMC Cancer 6, 107 16640785 10.1186/1471-2407-6-107 PMC1464148

[45] Lim do Y, Jeong Y, Tyner AL, et al. (2007) Induction of cell cycle arrest and apoptosis in HT-29 human colon cancer cells by the dietary compound luteolin. Am J Physiol Gastrointest Liver Physiol 292, G66–G75 16901994 10.1152/ajpgi.00248.2006

[46] Graudens E, Boulanger V, Mollard C, et al. (2006) Deciphering cellular states of innate tumor drug responses. Genome Biol 7, R19 16542501 10.1186/gb-2006-7-3-r19 PMC1557757

[47] Bromberg J (2002) Stat proteins and oncogenesis. J Clin Invest 109, 1139–1142 11994401 10.1172/JCI15617 PMC150969

[48] Niu G, Wright KL, Ma Y, et al. (2005) Role of Stat3 in regulating p53 expression and function. Mol Cell Biol 25, 7432–7440 16107692 10.1128/MCB.25.17.7432-7440.2005 PMC1190305

[49] Alas S & Bonavida B (2003) Inhibition of constitutive STAT3 activity sensitizes resistant non-Hodgkin's lymphoma and multiple myeloma to chemotherapeutic drug-mediated apoptosis. Clin Cancer Res 9, 316–326 12538484

[50] Ni Z, Lou W, Leman ES, et al. (2000) Inhibition of constitutively activated Stat3 signaling pathway suppresses growth of prostate cancer cells. Cancer Res 60, 1225–1228 10728680

[51] Konnikova L, Kotecki M, Kruger MM, et al. (2003) Knockdown of STAT3 expression by RNAi induces apoptosis in astrocytoma cells. BMC Cancer 3, 23 13678425 10.1186/1471-2407-3-23 PMC212316

[52] Kotha A, Sekharam M, Cilenti L, et al. (2006) Resveratrol inhibits Src and Stat3 signaling and induces the apoptosis of malignant cells containing activated Stat3 protein. Mol Cancer Ther 5, 621–629 16546976 10.1158/1535-7163.MCT-05-0268

[53] Opdenakker G & Van Damme J (2004) The countercurrent principle in invasion and metastasis of cancer cells. Recent insights on the roles of chemokines. Int J Dev Biol 48, 519–527 15349826 10.1387/ijdb.041796go

[54] Liotta LA & Kohn EC (2001) The microenvironment of the tumour–host interface. Nature 411, 375–379 11357145 10.1038/35077241

[55] Loberg RD, Bradley DA, Tomlins SA, et al. (2007) The lethal phenotype of cancer: the molecular basis of death due to malignancy. CA Cancer J Clin 57, 225–241 17626119 10.3322/canjclin.57.4.225

[56] Chen X, Beutler JA, McCloud TG, et al. (2003) Tannic acid is an inhibitor of CXCL12 (SDF-1alpha)/CXCR4 with antiangiogenic activity. Clin Cancer Res 9, 3115–3123 12912963

